# Suicide Attempts in Panic Disorder: Clinical Effects on Treatment Response and Link to Fear of Cognitive Dyscontrol

**DOI:** 10.1192/j.eurpsy.2023.457

**Published:** 2023-07-19

**Authors:** H. Kim, H.-Y. Jung, M. Bang, S.-H. Lee

**Affiliations:** ^1^Department of Psychiatry, CHA Bundang Medical Center; ^2^CHA University, Gyeonggi-do, Korea, Republic Of

## Abstract

**Introduction:**

Panic disorders (PD) are associated with suicidality. The link between PD and suicide has been suggested to be depression; however, this remains controversial. Comprehensive research on the history of suicide attempts (SAs) in patients with PD is scarce.

**Objectives:**

This study investigated the characteristics and pharmacological short- and long-term treatment responses of patients with PD, with or without SAs. Moreover, a network analysis was conducted to investigate the central symptoms and their connection to suicidality among SA-related variables with and without SAs.

**Methods:**

We investigated the characteristics of SAs in patients with PD using PD-related scales, magnetic resonance imaging, and network approaches. A total of 1151 subjects were enrolled, including 755 patients with PD (97 with SA and 658 without SA) and 396 healthy controls. Suicide and PD-related scales were also administered.

**Results:**

Our results revealed that the scores of all symptom severities were significantly higher in the PD+SA group than in the PD-SA group. Multiple linear regression analysis revealed that short- and long-term pharmacological treatment responses were significantly poor in the PD+SA group. Network analysis showed that fear of cognitive dyscontrol (FCD) was the strongest central symptom among strength, expected influence (1 and 2 step), randomized shortest path betweenness, and eigenvector centrality measures in PD+SA, whereas depression was the central symptom in PD-SA.

**Table 1.** Results of multiple regression analysis to predict treatment response for patients with panic disorder.

	Treatment response at 8 weeks (*n =* 450)	[R^2^ =0.19]	Treatment response at 6 month (*n =* 379)	[R^2^ =0.20]	Treatment response at 1 year (*n =* 329)	[R^2^ =0.22]
	*Β*	*p*-value	*β*	*p*-value	*β*	*p*-value
Gender	0.10	0.15	0.14	0.09	0.08	0.39
Age	-0.06	0.44	0.04	0.62	0.01	0.94
Baseline PDSS total score	0.46	<0.001**	0.48	<0.001**	0.42	<0.001**
Baseline BDI-II total score	0.05	0.65	0.09	0.47	0.17	0.19
Baseline PSWQ total score	-0.07	0.47	-0.11	0.29	-0.18	0.09
Baseline ASI-R total score	-0.14	0.19	-0.19	0.10	-0.19	0.11
Baseline ETISR-SF total score	0.07	0.34	0.04	0.60	0.11	0.24
A history of the suicide attempt	-0.19	0.01*	-0.20	0.02*	-0.28	0.002*

*Note*: Model *p*-values <0.001.

* *p* < 0.05.

** *p* < 0.001.

Abbreviations: PD, panic disorder; SA, suicide attempt; PDSS, Panic Disorder Severity Scale; BDI-II, Beck Depression Inventory-II; PSWQ, Penn State Worry Questionnaire; ASI-R, Anxiety Sensitivity Inventory-Revised; ETISR-SF, The Early Trauma Inventory Self Report-Short Form.

**Image:**

**Figure:**
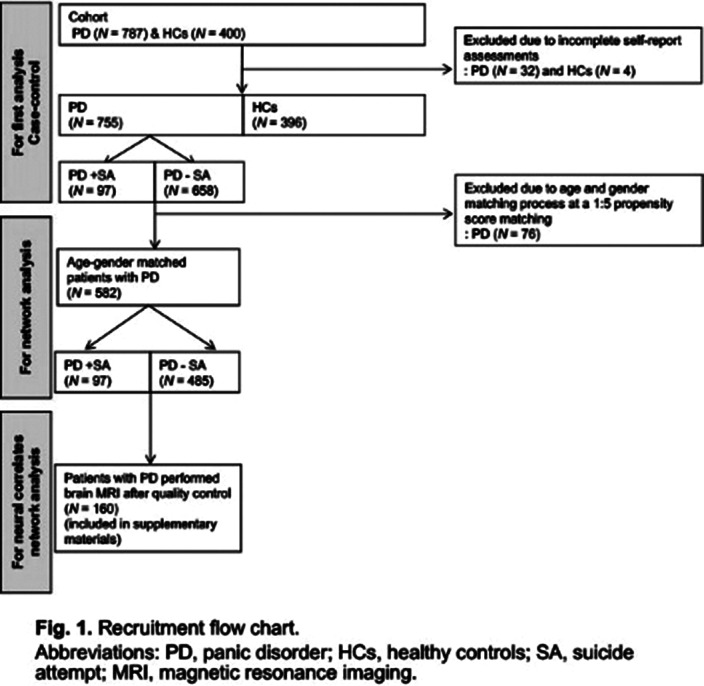

**Image 2:**

**Figure:**
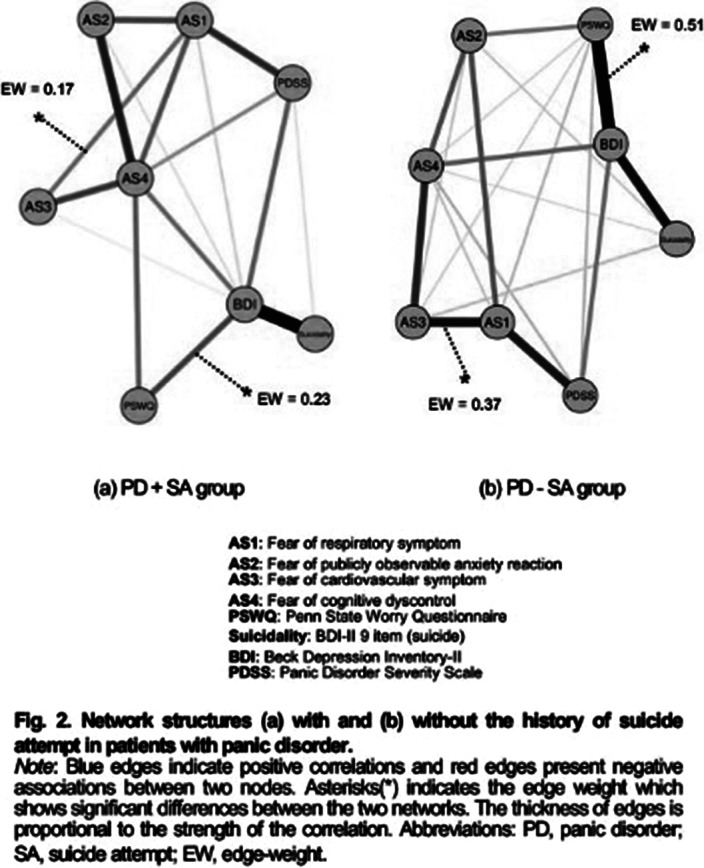

**Image 3:**

**Figure:**
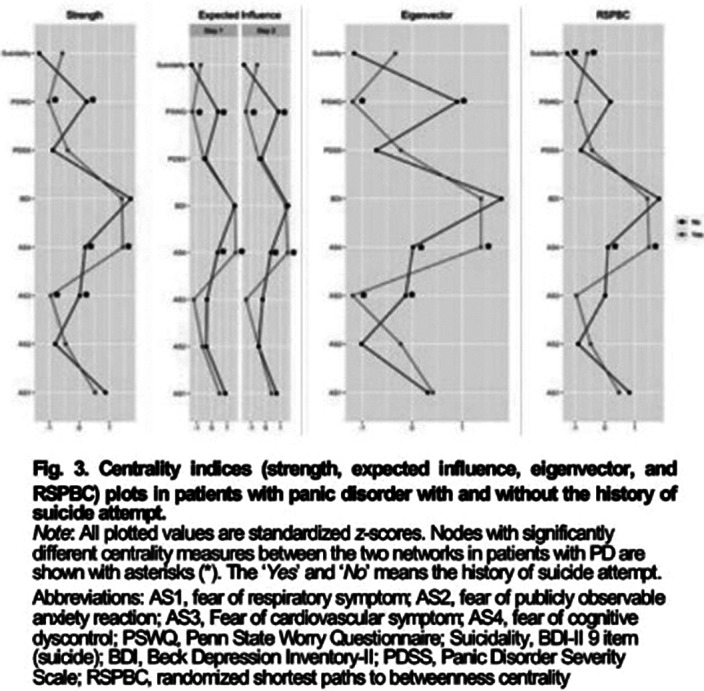

**Conclusions:**

Our results suggest that SA history could be associated with high symptom severity and poor pharmacological treatment response in patients with PD and that FCD is the central symptom in the PD+SA network.

**Disclosure of Interest:**

None Declared

